# Retraction Note: M2 macrophagy-derived exosomal miRNA-5106 induces bone mesenchymal stem cells towards osteoblastic fate by targeting salt-inducible kinase 2 and 3

**DOI:** 10.1186/s12951-024-02527-z

**Published:** 2024-05-12

**Authors:** Yuan Xiong, Lang Chen, Chenchen Yan, Wu Zhou, Tao Yu, Yun Sun, Faqi Cao, Hang Xue, Yiqiang Hu, Dong Chen, Bobin Mi, Guohui Liu

**Affiliations:** 1 Department of Orthopaedics, Union Hospital, Tongji Medical College, Huazhong University of Science and Technology, Wuhan, 430022 China; 2 Department of Orthopedic Surgery, Tongji Hospital, Tongji University School of Medicine, Shanghai, 200065 China; 3 Department of Neurosurgery, Union Hospital, Tongji Medical College, Huazhong University of Science and Technology, Wuhan, 430022 China

**Journal of Nanobiotechnology (2020) 18:66**

10.1186/s12951-020-00622-5

The authors have retracted this article. After publication, the authors found a duplicated image in Fig. 2e, which was addressed by a Correction [1]. However, further concerns were raised regarding highly similar images in Fig. 5 h (antagomiR-NC, right) and 6n (siRNA-NC, right).

The authors have thoroughly checked the original data, and determined that there were oversights during data collection. Therefore, they have decided to retract the article to avoid misleading the readers.

Lang Chen, Tao Yu, Hang Xue, Faqi Cao, Dong Chen, Bobin Mi and Guohui Liu agree to this retraction. Yuan Xiong, Chenchen Yan, Wu Zhou, Yun Sun and Yiqiang Hu have not responded to any correspondence from the Publisher about this retraction.

## References

[1] Xiong Y, Chen L, Yan C (2021). Correction to: M2 macrophagy-derived exosomal miRNA-5106 induces bone mesenchymal stem cells towards osteoblastic fate by targeting salt-inducible kinase 2 and 3. J Nanobiotechnol.

